# Atypical sound discrimination in children with ASD as indicated by cortical ERPs

**DOI:** 10.1186/s11689-017-9194-9

**Published:** 2017-04-05

**Authors:** Aurélie Bidet-Caulet, Marianne Latinus, Sylvie Roux, Joëlle Malvy, Frédérique Bonnet-Brilhault, Nicole Bruneau

**Affiliations:** 1 UMR Inserm U930, Université François Rabelais de Tours, Tours, France; 2 Brain Dynamics and Cognition Team, Lyon Neuroscience Research Center; CRNL, INSERM U1028, CNRS UMR5292, University of Lyon 1, Lyon, France; 3 CHRU de Tours, Service de Pédopsychiatrie, Tours, France

**Keywords:** Voice, FTPV, Speech, Autism, Development, Auditory

## Abstract

**Background:**

Individuals with autism spectrum disorder (ASD) show a relative indifference to the human voice. Accordingly, and contrarily to their typically developed peers, adults with autism do not show a preferential response to voices in the superior temporal sulcus; this lack of voice-specific response was previously linked to atypical processing of voices. In electroencephalography, a slow event-related potential (ERP) called the fronto-temporal positivity to voice (FTPV) is larger for vocal than for non-vocal sounds, resulting in a voice-sensitive response over right fronto-temporal sites. Here, we investigated the neurophysiological correlates of voice perception in children with and without ASD.

**Methods:**

Sixteen children with autism and 16 age-matched typically developing children heard vocal (speech and non-speech) and non-vocal sounds while their electroencephalographic activity was recorded; overall IQ was smaller in the group of children with ASD. ERP amplitudes were compared using non-parametric statistical tests at each electrode and in successive 20-ms time windows. Within each group, differences between conditions were assessed using a non-parametric Quade test between 0 and 400 ms post-stimulus. Inter-group comparisons of ERP amplitudes were performed using non-paired Kruskal-Wallis tests between 140 and 180 ms post-stimulus.

**Results:**

Typically developing children showed the classical voice-sensitive response over right fronto-temporal electrodes, for both speech and non-speech vocal sounds. Children with ASD did not show a preferential response to vocal sounds. Inter-group analysis showed no difference in the processing of vocal sounds, both speech and non-speech, but significant differences in the processing of non-vocal sounds over right fronto-temporal sites.

**Conclusions:**

Our results demonstrate a lack of voice-preferential response in children with autism spectrum disorders. In contrast to observations in adults with ASD, the lack of voice-preferential response was attributed to an atypical response to non-vocal sounds, which was overall more similar to the event-related potentials evoked by vocal sounds in both groups. This result suggests atypical maturation processes in ASD impeding the specialization of temporal regions in voice processing.

## Background

As initially described by Kanner [1], autism is characterized by two core features: (i) impairments in social interactions and abnormal development of verbal and non-verbal communication and (ii) repetitive and ritualized behaviors associated with a restricted range of interests [2]. Recently, and consistent with Kanner’s first observations, abnormal reactivity to sensory stimulations, including sounds, has been introduced in the diagnostic criteria of autism in the DSM-5 (Diagnostic and Statistical Manual of Mental Disorders, 5th edition, [2]), highlighting atypical processing of environmental sounds. Kanner also emphasized the relative indifference of his patients to the human voice. He writes of one of his patients, “He did not register any change of expression when spoken to,” and of another, “He did not respond to being called or to any other words addressed to him.” Indeed, a striking characteristic of children with autism is their poor orienting to the human voice [3–5]. For example, when given a choice between their mothers’ speech and a mixture of environmental noises, children with autism either show a lack of orientation for either sound or an active interest in environmental noises only [3, 6]. Experimental investigations using event-related potentials (ERPs) and an oddball paradigm have shown that, in contrast to typically developed (TD) children, children with autism spectrum disorder (ASD) aged 3–4 years [7] or aged 6–12 years [8] do not automatically orient their attention to vocal stimuli. In addition, a brain-imaging study using functional magnetic resonance imaging (fMRI) showed no difference in brain activation to voice (speech and non-speech) and to non-voice stimuli in adults with ASD, mainly attributed to a decreased response to vocal sounds [9]; this result has recently been refuted on a larger sample of ASD participants without accompanying intellectual impairment [10]. Taken together, these results suggest atypical processing of voice stimuli in patients with ASD, which could be central to the deficits in social interaction and communication.

This lack of voice-sensitivity in ASD contrasts with the observation of “voice-sensitive areas”—that is, brain regions that are more activated by vocal than non-vocal stimuli—that have been identified along the upper bank of the superior temporal sulcus (STS) in healthy adults, with greater sensitivity on the right than on the left hemisphere, in several fMRI studies [11–14]. Using electroencephalography (EEG) or magnetoencephalography (MEG), a voice-sensitive response, discriminating between vocal (speech and/or non-speech) and non-vocal stimuli, was identified at fronto-temporal sites, predominantly over the right hemiscalp, in an early latency range between 100 and 300 ms after stimulus onset [15–18]. The voice-sensitive response was mainly driven by a fronto-temporal positivity to voice (FTPV; [19]), a slow event-related potential larger to vocal than non-vocal sounds and thought to reflect the activation of the “voice-selective areas” [17, 18]. Importantly, automatic voice processing has been identified in typically developing children and infants from a very early age. Studies using fMRI and near infrared spectroscopy (NIRS) have suggested that the voice-sensitive brain systems emerged between 4 and 7 months of age [20–22]. Using EEG, the FTPV was found to begin as early as 60 ms after stimulus onset, in 4- to 5-year-old typically developing children, passively hearing human vocal and environmental sounds [19].

The FTPV occurs at the latencies of the successive peaks of the typical auditory response elicited either at temporal or at fronto-central sites. Indeed, child auditory-evoked potential waveforms are characterized by a large response recorded temporally, the T-response, clearly dissociated from successive positive-negative fronto-central responses peaking, respectively, around 100 (P100) and 200 ms (N250) [23, 24]. The temporal response, prominent in children, consists of three successive deflections: a first negative peak, the Na or N1a; followed by a positive deflection, named Ta; and finally a negative deflection, the Tb or N1c [23–28]. The biphasic Ta-Tb response was initially described in adults and named the T-complex [29–31] while others referred to them as N1a and N1c peaks afterwards [32, 33]; in the current manuscript, we consider all three deflections and refer to them as the “T-response.” The FTPV overlaps the T-response typically recorded over bilateral sites within the first 300 ms following the presentation of a non-vocal sound; therefore, the T-response appears reduced for vocal sounds [19]. On the contrary, the fronto-central response is not voice sensitive [19]. The T-response to pure tones has been shown to be atypical in children with ASD [28, 34] and linked to communicative impairments.

Therefore, the aim of the present study was to investigate voice processing in children with ASD aged 7–12 years by comparing cortical ERPs to vocal sounds (both non-speech and speech sounds) and non-vocal sounds. We hypothesized that, contrary to age-matched typically developing children, children with ASD will not differentially process vocal and non-vocal stimuli. However, whether this lack of sensitivity is related to atypical voice processing or atypical processing of non-vocal sounds remains an open question.

## Methods

### Participants

Sixteen children with ASD (15 boys and 1 girl) aged from 7 years 8 months to 12 years 2 months (mean age ± standard deviation 10 years 6 moths ± 1 year 5 months) participated in the study. They were recruited from the Child Psychiatry Centre of the University Hospital of Tours. Children with neurological disorders (including seizures), physical abnormalities, neurologic impairment in motor or sensory function, or genetically defined disorders were excluded. All had normal hearing, verified by subjective or objective (when necessary) audiometric tests performed before ERP recordings.

Diagnosis of ASD was made by experienced clinicians according to DSM-IV-R criteria [35] at the time of electrophysiological recordings and using the Autism Diagnostic Observation Schedule-Generic (ADOS-G; [36]) and/or the Autism Diagnostic Interview-Revised (ADI-R; [37]). Developmental quotients (DQs) were assessed by the Echelles Différentielles d’Efficiences Intellectuelles (EDEI-R; [38]) or the Wechsler Intelligence Scale for Children (WISC III and WISC IV). These two developmental scales provide verbal developmental (vDQ; mean ± SD 69 ± 25) and non-verbal developmental (nvDQ; mean ± SD 85 ± 18) quotients.

Sixteen typically developing (TD) children (15 boys and 1 girl, mean age ± standard deviation 10 years 5 months ± 1 year 5 months) were matched in age and gender with the patients. All typically developed children had normal education level and language development. The Ethics Committee of the University Hospital of Tours approved the protocol (Comité de Protection des Personnes (CPP) Tours Ouest 1; n°2006-R5). Signed informed consent was obtained from parents, and assent was given by the children.

### Paradigm

#### Stimuli

The stimuli used were sounds extracted from the vocal and non-vocal sequences used in Belin et al.’s block-design fMRI studies [11, 13] (http://vnl.psy.gla.ac.uk/resources.php). The vocal sounds, both non-speech (Voc^NSp^) (e.g., laughing, sighing, and coughing) and speech (Voc^Sp^) (syllables in several languages, e.g., English, Finnish, Arabic), were produced by a large number of speakers of both genders and of different ages. No French words were included in the experiment so as to prevent the influence of linguistic processing on the ERPs because of inherent differences in language development across the two groups. Overall, there were 53 unique vocal non-speech sounds and 67 unique vocal speech sounds. Non-vocal sounds (NVoc) consisted of sounds from a wide variety of sources, including the human environment (such as telephones, alarms, cars), musical instruments (such as bells and orchestral instruments), and nature (such as streams, wind, animal sounds); in total, there were 160 unique non-vocal sounds. Sound duration was adjusted to 500 ms, and an envelope of 50 ms decay time was applied to the end of each sound to minimize clicks at sound offset. All sounds were normalized according to the root mean square of their amplitude.

The stimuli used in this ERP study were previously selected in a pretest session performed with 4- to 5-year-old typically developing children; see [19].

**Fig. 1:**
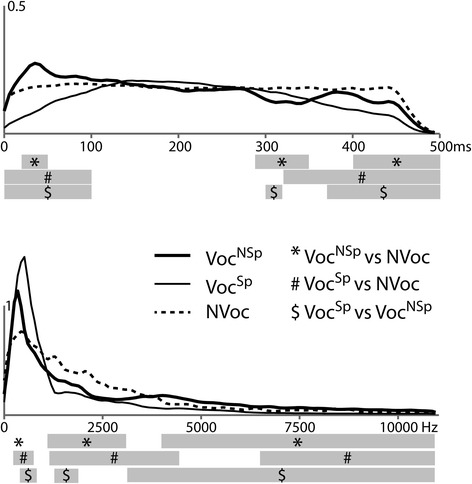
Acoustical differences between sound categories. **a** Power analysis in time: average power of each sound category over the 500 ms sound duration. **b** Power analysis in frequency: average power spectrum of each sound category. Statistical differences between sound categories are indicated by *gray bars*

To better characterize the sound categories at the acoustic level, analyses of sound power in the temporal and spectral domains were performed at each time or frequency bin (11.6 ms; 43 Hz) using a statistical test based on randomization [39] and controlling the false discovery rate to correct for multiple comparisons. Randomization consisted of (1) the random constitution of the two samples to compare, (2) the sum of squared sums of values in the two obtained samples, and (3) the computation of the difference between these two statistic values. We performed 50,000 such randomizations to obtain an estimate of the distribution of this difference under the null hypothesis. From this distribution, we estimated the threshold corresponding to a significant difference between two conditions; this threshold was then compared to the empirical difference between the values in the two conditions. This analysis highlighted significant differences between sound categories both in frequency and time domains (see Fig. 1).

#### Design

Stimuli were delivered with Presentation® (Neurobehavioral Systems Inc.), through two loudspeakers placed 1.20 m in front of the subject at approximately 10° on both sides of the interaural axis. Overall stimulus intensity was adjusted to 70 dB SPL at the subject’s head. Stimuli were presented with a constant interstimulus interval (offset to onset) of 700 ms.

Two successive sequences were delivered in alternating order between subjects. A sequence comprised one block of stimulation repeated three times. In the vocal sequence, 582 vocal stimuli, 44% non-speech, were presented as standard and non-vocal sounds as deviants (15%). In the non-vocal sequence (NVoc), 582 non-vocal stimuli were presented as standard and vocal sounds as deviants (15%). The present study only reports ERPs recorded to standard stimuli, i.e., Voc^NSp^ and Voc^Sp^ in the first sequence and NVoc in the second sequence.

### EEG recording

During the recording session, children sat in a comfortable armchair in a dimly lit, soundproofed room and watched a silent video of their choice.

Auditory event-related potentials were collected using the NeuroScan electrophysiological data acquisition system (SCAN 4.3). In all TD children and in 11 children with ASD, the electroencephalogram was recorded from 28 Ag/AgCl scalp electrodes referenced to the nose. Five children with ASD could only tolerate the placement of 11 scalp electrodes: Fz, Cz, Pz, F7, F8, T7, T8, T5, and T6 placed according to the International 10-20 System and M1 and M2 on the left and right mastoid sites, respectively. Vertical electrooculogram (EOG) activity was recorded from electrodes placed above and below the right eye. All electrode impedance levels were kept below 10 kΩ. The EEG and EOG were amplified with an analog band-pass filter (0.3–70 Hz; slope 6 dB/octave) and sampled at 500 Hz.

### Data analysis

EEG data were preprocessed within NeuroScan (Compumedics Inc., Neuroscan, 2003). Epochs recorded in response to standard stimuli immediately following a deviant stimulus (i.e., 102 trials) were excluded from the analyses in order to focus on responses to frequent standard stimuli. Epochs corresponding to responses to specific stimuli, e.g., stimuli deemed ambiguous a posteriori (corresponding to 144 trials in the vocal sequence), and animal vocalization (57 trials in the non-vocal sequence) were excluded from the analysis. Animal vocalizations were excluded as they have the same physiological origin as human vocal sounds. Automatic correction of eye movements was then applied. Eye-movement artifacts were eliminated using a spatial filter transform developed by NeuroScan (Compumedics Inc., Neuroscan, 2003). The spatial filter is a multi-step procedure that generates an average eye blink, utilizes a spatial singular value decomposition based on principal component analysis (PCA) to extract the first component and covariance values, and then uses those covariance values to develop a filter that retains the EEG activity of interest. EEG periods with movement artifacts were manually rejected. After rejection, the averaged numbers of trials (±SEM) were 93 ± 14, 120 ± 18, and 274 ± 37 in children with ASD and 108 ± 17, 138 ± 22, and 321 ± 49 in TD children, for Voc^NSp^, Voc^Sp^, and NVoc, respectively. There were significantly fewer trials in ASD children than in TD children in each condition (*p* < 0.05) as assessed using Kruskal-Wallis tests. However, in all conditions, the average number of trials in the ERPs was always greater than 90, which is sufficient to accurately measure auditory ERPs in children. For each subject and each stimulus type, ERPs were averaged and baseline corrected according to a 100 ms prestimulus period. A digital zero-phase-shift low-pass filter (30 Hz) was then applied. ERPs were analyzed with the ELAN® software [40]. As indicated in previous electrophysiological data [15, 41], responses specifically elicited by voice compared to non-voice occur early; the analysis therefore focused on the first 400 ms after stimulus onset.

Scalp ERP topographies displayed in Fig. 4 were generated using a two-dimensional spherical spline interpolation [42] and a radial projection from Cz (top views), which respects the length of the meridian arcs [42, 43].

### Statistical analysis

To limit assumptions regarding the data distribution, non-parametric statistical tests were used to compare ERP amplitudes. Differences between conditions (Voc^NSp^, Voc^Sp^, and NVoc) within each group were assessed using a non-parametric Quade test at each of the 11 electrodes and in successive 20-ms time windows between 0 and 400 ms post-stimulus. In order to correct for multiple comparisons, results are reported at threshold corresponding to *p* < 0.00025 (Bonferroni correction according to the number of tested electrodes and time-windows). Two-by-two post hoc comparisons were assessed using a non-parametric paired Wilcoxon test at the significant electrodes and time windows found with the Quade test.

Inter-groups comparisons of Voc^NSp^, Voc^Sp^, and NVoc ERP amplitudes were performed using non-parametric non-paired Kruskal-Wallis tests at each of the 11 electrodes and in successive 20-ms time windows between 140 and 180 ms post-stimulus. The 140–180-ms time window corresponds to the significant voice effect at right fronto-temporal electrode sites in TD children.

## Results

**Fig. 2:**
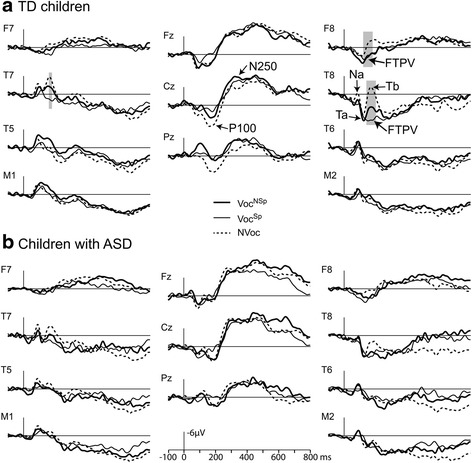
ERPs evoked by vocal sounds (either non-speech: Voc^NSp^, or speech: Voc^Sp^) and non-vocal sounds (NVoc) at fronto-temporal, temporo-mastoïd, and central electrode sites in TD children (**a**) and children with ASD (**b**). Significant differences between conditions according to the Quade test (after Bonferroni correction) are indicated by *gray bars*

Figure 2 presents the grand-averaged ERPs to Voc (either Voc^NSp^ or Voc^Sp^) and NVoc stimuli recorded from the 11 electrode sites in the two groups of children. Visual inspection of the grand average shows that ERP waveforms varied according to conditions much more in TD children than in children with ASD. This was particularly striking over right fronto-temporal sites (F8 and T8) and was confirmed by results of statistical analyses.

### TD children

ERPs elicited by the three stimulus categories over fronto-central midline sites (Fig. 2a) displayed similar waveforms with two successive positive-negative deflections peaking at around 200 and 400 ms, respectively, with polarity reversal at mastoid sites, in particular on the left side. ERPs recorded over fronto-temporal sites displayed different waveforms according to stimulus conditions. This was particularly marked at right temporal electrodes (T8): NVoc stimuli evoked the T-response whose peaks culminated at around 80, 120, and 165 ms. This T-response was different in response to Voc stimuli with reduced (for Voc^NSp^ stimuli) or absent (for Voc^Sp^ stimuli) Tb peak and more positive preceding peaks (Na and Ta). At the right frontal F8 electrode, ERPs to Voc appeared as a more sustained positive deflection than the response to NVoc stimuli, suggesting that Voc stimuli elicited a positive slow wave, better seen at the right fronto-temporal F8 electrode than at the temporal T8 site where it overlaps the T-response. This positive slow wave previously observed in adults [15–18] and 4- to 5-year-old children [19] corresponds to the FTPV.

Statistical analyses using Quade tests (Bonferroni corrected *p* < 0.00025) indicated significant differences between ERPs evoked by the different stimulus categories over right fronto-temporal sites (F8 120–180 ms, T8 140–200 ms) and over a left temporal site (T7 160–180 ms). Post hoc Wilcoxon tests (*p* < 0.05) indicated that over right fronto-temporal sites, Voc^NSp^ and Voc^Sp^ sounds elicited a greater positivity than NVoc stimuli; this positivity was greater to Voc^Sp^ than to Voc^NSp^ at T8. Over T7 (160–180 ms), Voc^Sp^ sounds elicited a larger positivity than Voc^NSp^ and NVoc stimuli.

In summary, a significant FTPV was observed over right fronto-temporal sites in response to vocal speech and non-speech stimuli in comparison to non-vocal sounds in 7–12-aged TD children.

### Children with ASD

As shown on Fig. 2b, ERP waveforms were rather similar for the three conditions in children with ASD at all recorded electrodes. ERPs elicited at fronto-central midline sites displayed the two successive positive-negative deflections also found in TD children. Over temporal sites, irrespective of the stimulus condition, ERPs showed a small Na deflection peaking at around 75 ms, followed by a large slow positive deflection.

Quade tests indicated no significant stimulus-related differences between ERPs to Voc^NSp^, Voc^Sp^, and NVoc sounds. Therefore, in 7–12-aged children with ASD, responses to vocal and non-vocal sounds did not seem to differ as much as in TD children.

### Children with ASD vs. TD children

**Fig. 3:**
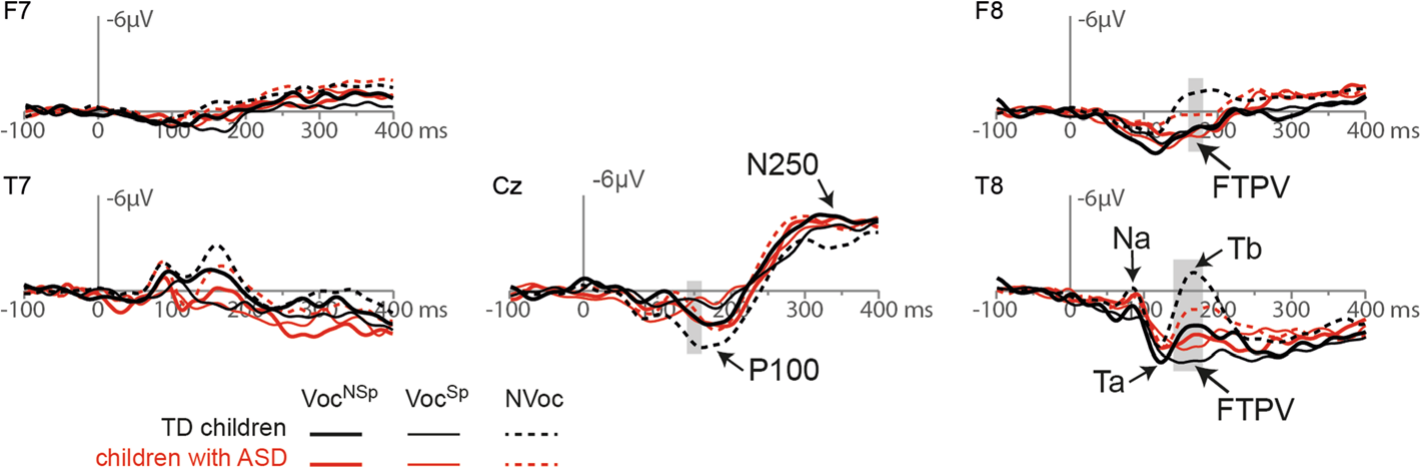
ERPs evoked by vocal sounds (either non-speech: Voc^NSp^, or speech: Voc^Sp^) and non-vocal sounds (NVoc) at left and right frontal (F7 and F8, respectively), temporal (T7 and T8, respectively), and central (Cz) electrode sites in TD children (*black lines*) and children with ASD (*red lines*). Significant differences (*gray bars*) between children with ASD and TD children were only found for the NVoc sounds

**Fig. 4:**
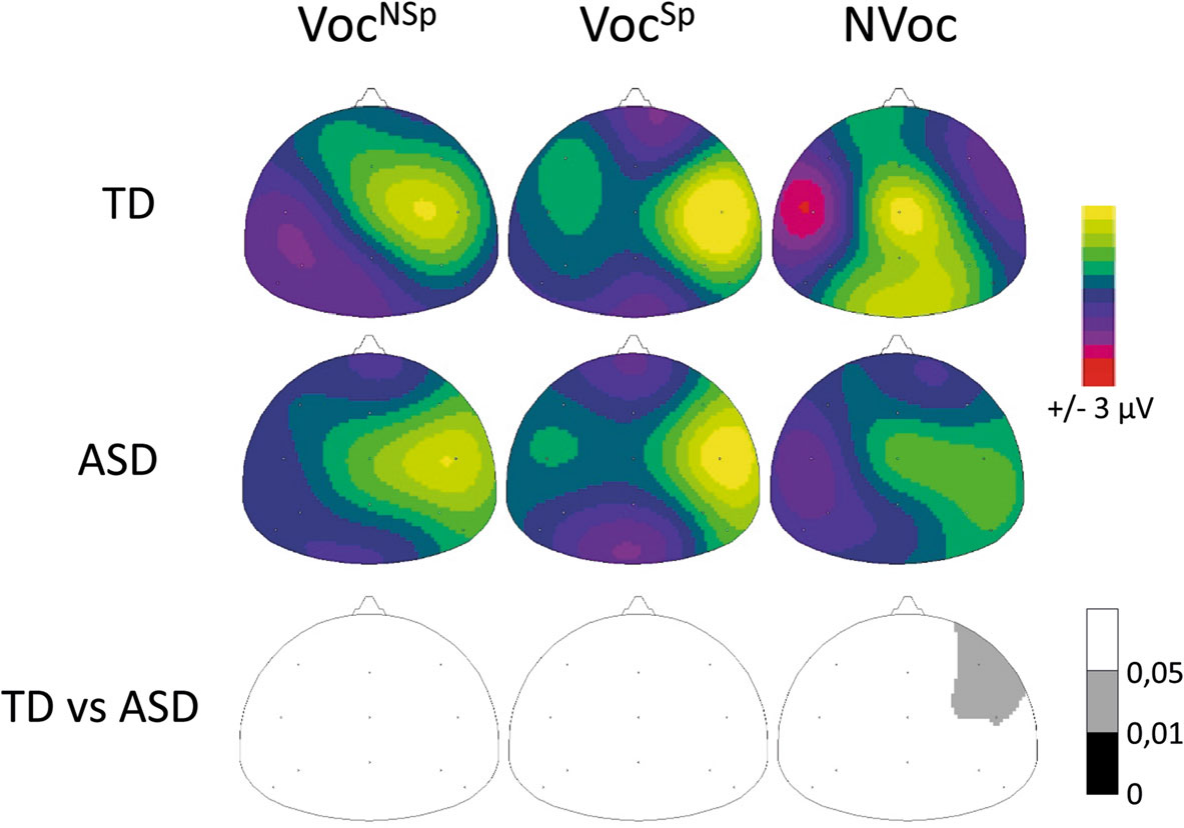
Scalp potential distributions (*top view*) of responses to vocal (either non-speech: Voc^NSp^, or speech: Voc^Sp^) and non-vocal (NVoc) stimuli in the 140–180-ms time window in 16 children with ASD (*middle row*) and 16 age- and gender-matched TD children (*top row*). The 140–180-ms time window corresponds to the significant voice effect at right fronto-temporal electrode sites in TD children. *Bottom row*: topographies of the *p* value resulting from the group comparison between TD and ASD children for each type of stimuli between 140 and 180 ms

Group differences were significant for ERPs to NVoc sounds within the significant time window of the FTPV in TD children (140–180 ms). Children with ASD presented a smaller P100 at a central site (Cz 140–160 ms) and a smaller right fronto-temporal negative Tb peak (T8 140–180 ms, F8 160–180 ms) than TD children (*p* < 0.05, Figs. 3 and 4). No significant difference was found, between ASD and TD children, in the amplitude of ERPs to Voc^Sp^ and Voc^NSp^ sounds.

## Discussion

The aim of this study was to investigate voice processing in children with ASD. To that aim, we recorded brain activity evoked by vocal and non-vocal sounds in children with ASD and age-matched typically developed children. The results showed differences in the response to vocal and non-vocal sounds in TD children but not in children with ASD. Comparison across groups revealed significant differences in the response to non-vocal sounds but not to vocal sounds, highlighting that the lack of difference was attributable to atypical processing of non-vocal sounds.

### Processing of vocal and non-vocal stimuli in TD children

In TD children aged 7 to 12 years, non-vocal stimuli elicited the classical T-response, as previously observed in younger TD children aged 4–5 years in response to the same non-vocal sounds [19]. In contrast, vocal stimuli elicited a different pattern over temporal sites with a flattened Tb peak. This result is consistent with a study showing a modulation of Tb amplitude by “speechness” [26]. A thorough observation of the Tb peak reported in studies using speech-like sounds [27] to those using other non-speech sounds [23–25] suggests a similar modulation of the Tb peak by “voiceness.”

We suggest that this flattened T-response to vocal sounds is due to a superimposed positive slow wave spreading over right fronto-temporal sites. This slow wave corresponds to the fronto-temporal positivity to voice (FTPV) previously described in younger children [19] and adults, e.g., [15, 41]; it may also correspond to the early part of the lateral anterior positivity (LAP) that has been associated with the analysis of discourse [44, 45]. The T-response was especially flattened for vocal speech sounds, as illustrated by significant differences within the voice category over bilateral temporal sites in the latency range of the Tb peak. Vocal non-speech sounds elicited a right-lateralized FTPV while speech stimuli elicited a bilateral FTPV. This is consistent with previous findings in younger children [19] and in adults since De Lucia et al. [18] and Bruneau et al. [15] described a right-lateralized FTPV to vocal non-speech sounds whereas the FTPV was recorded bilaterally in studies using both vocal speech and non-speech stimuli [16, 17]. ERP differences between both vocal and non-vocal sounds are unlikely to be accounted for by acoustic differences between the stimuli. Before 100 ms, the acoustic power of non-vocal sounds fits in between the acoustic powers of the two vocal sound categories (Fig. 1). Moreover, acoustic differences are also present between the two vocal sound categories, although they evoked similar ERPs.

Previous studies suggested that the FTPV corresponds to the activation of the temporal voice areas described in fMRI studies with adult participants, bilaterally localized in the anterior STS with a right hemisphere predominance [11–13, 46–50]. Consistent with our observations, activity linked to processing the non-verbal aspect of vocal stimuli (e.g., vocal non-speech sounds) is also lateralized to the right hemisphere [13, 47]. Similarly, an fMRI study with 7-month-old infants showed a preferential response to non-speech vocal sounds in the right anterior temporal cortex [20]. Thus, based on the literature [19], we hypothesize that the FTPV observed in children also reflects the activation of the temporal voice areas, although this should be explored with more appropriate methods than the low-density montage used here and non-vocal stimuli matched in spectro-temporal complexity.

The FTPV, as the early LAP, seems to reflect the processing of “voiceness.” This initial analysis of “voiceness” may feed into the late LAP proposed to reflect discourse processing [44].

### Processing of vocal and non-vocal stimuli in children with ASD

In contrast to the ERP results found in TD children, children with ASD display few stimulus-related ERP differences. The lack of difference between the ERPs evoked by vocal and non-vocal sounds in ASD children could relate to changes in sensitivity to stimulus-type differences, sustained attention, or developmental quotient. In the current study, most of the children with ASD presented accompanying intellectual disabilities; in order to include these children in the protocol, a passive listening task was used. This ensured that differences in developmental quotient did not influence task comprehension and execution. Participants were not required to perform a task with the auditory stimuli. They were watching a silent video so as to reduce potential confounds in the allocation of attentional resources. It has been shown that TD children automatically attend to speech stimuli, even when watching silent videos, albeit to a smaller extent than when explicitly directing their attention toward the auditory modality [51]. On the contrary, this automatic orientation to speech stimuli appears reduced in children with specific language impairment [51]. Another study has demonstrated that children with ASD showed impaired attention orienting to speech sounds, associated with intact sensory processing [8]. However, an impaired attention orienting is unlikely to explain our results since, in the present study, group differences were only observed for non-vocal sounds. The most likely explanation of these differences is that participants with ASD are differently sensitive to stimulus-type differences. Children with autism present abnormal responses to all sounds, both at behavioral [1, 52] and neural levels [28, 34]. An abnormal response to sounds could hinder their processing and prevent the discrimination between vocal and non-vocal sounds. This finding is in agreement with a previous fMRI study performed with similar stimulations in adults with ASD, showing no difference in brain activation between voice and non-voice stimuli [9], although it contradicts more recent evidence in adults with ASD with no intellectual disabilities [10]. These fronto-temporal responses are clearly dissociated from the fronto-central response, which appears as a prominent positivity in children around 100–200 ms. The fronto-central response to non-vocal sounds differed between TD children and children with ASD, although it does not discriminate vocal from non-vocal sounds in TD children [19], again highlighting atypical processing of non-vocal sounds.

Interestingly, the present results clearly showed that the ERPs elicited by vocal stimuli are strongly similar between children with ASD and TD children. On the contrary, ERPs to non-vocal stimuli greatly differ in children with ASD and in TD children over central and right fronto-temporal sites. The present results are in contradiction to previous fMRI findings showing, in five adult patients with ASD, similar responses to non-vocal sounds in TD adults and adults with ASD, but different activations to vocal sounds (speech and non-speech), bilaterally along the upper bank of the STS [9]. This discrepancy could arise from the ASD populations tested (e.g., with or without accompanying intellectual impairment), the sample size (only five adults in the fMRI study), and the age of the participants (adults vs. children). This discrepancy echoes data from the face perception literature [53–55]. While children with ASD between 4 and 5 years old showed typical patterns of amplitude in ERPs to facial stimuli and an abnormal response to visual objects [53], in adult participants, group differences between ASD and TD participants were observed mainly in the response to faces [54]. Accordingly, while ERP amplitude to facial emotion differed between ASD and TD adults [55], no group differences were observed in children [55, 56]. Hence, the difference between studies with adults with ASD and children with ASD appear to reflect genuine maturational processes, rather than methodological differences.

Models of the development of face processing argue that brain regions involved in face processing are not initially face-specific and are activated by a broad range of stimuli; with time, their activity tunes to the type of stimuli mostly seen in the environment, e.g., upright faces, and yield to face-specific activity [57, 58]. Accordingly, we propose that prolonged experience with vocal stimuli during typical development allows the development of “filters” which in time permit the not-initially voice-sensitive regions of the STS to tune their activity toward processing voices. These filters would be derived from the acoustical information present in vocal sounds and may correspond to the voice configuration [59]. Consistent with this hypothesis, it has been shown that the underlying components of the T-response to a vowel sound maturate at different rates with the Ta and Tb peaks still not being fully mature at age 8 [60]. This multi-step maturation of the T-response might reflect the development of the “filters” that allows optimizing voice processing. In contrast, the development of these “filters” would be impaired in autism and the STS would not tune to a specific sound category resulting in a lack of specialization in processing voices. Consequently, the activity of the STS would also be strong for non-vocal sounds in children with ASD, resulting in the observation of slow wave components on fronto-temporal electrodes in response to non-vocal sounds; this would in turn yield to a flattened T-response and an absence of stimulus-type-related difference. This observation is consistent with some theories of autism such as the social motivation theory [61] or the weak central coherence (WCC)/enhanced perceptual functioning (EPF) [62, 63]. The social motivation theory stipulates that ASD results from an imbalance in attending to social and non-social stimuli, while the WCC and EPF theories propose that ASD is the consequence of atypical processing of stimuli, resulting from an imbalance between local and global processing. These theories are not mutually exclusive, and whether one or the other yields the current results remains to be tested. Nonetheless, recent evidence points toward impaired global processing of vocal sounds, as people with ASD fail to combine the acoustic features into a coherent percept [52, 64].

## Conclusions

Children with ASD displayed a similar response to vocal and non-vocal sounds over right fronto-temporal sites; therefore, they did not show a voice-preferential response. This lack of difference appears to be driven by an atypical processing of non-vocal sounds.

## Abbreviations

ADI-R: Autism Diagnostic Interview-Revised
ADOS: Autism Diagnostic Observation Schedule
ASDs: Autism spectrum disorders
DQs: Developmental quotients
DSM-5: Diagnostic and Statistical Manual of Mental Disorders, 5th edition
EDEI-R: Echelles Différentielles d’Efficiences Intellectuelles
EEG: Electroencephalography
ERP: Event-related potential
fMRI: Functional magnetic resonance imaging
FTPV: Fronto-temporal positivity to voice
MEG: Magnetoencephalography
nvDQ: Non-verbal developmental quotient
NVoc: Non-vocal sounds
TD: Typically developed
vDQ: Verbal developmental quotient
Voc^NSp^: Vocal non-speech sounds
Voc^Sp^: Vocal speech sounds
WISC: Wechsler Intelligence Scale for Children
